# Differences in Venous Thromboembolism Prevention and Outcome between Hospitalized Patients with Solid and Hematologic Malignancies

**DOI:** 10.1055/s-0039-1692203

**Published:** 2019-05-30

**Authors:** Rocío Figueroa, Ana Alfonso, María Marcos, José María López-Picazo, Alberto García-Mouriz, Ignacio Gil-Bazo, José Rifón, José Hermida, José Antonio Páramo, Ramón Lecumberri

**Affiliations:** 1 Hematology Service, Clínica Universidad de Navarra, Pamplona, Navarra, Spain; 2 Department of Oncology, Clínica Universidad de Navarra, Pamplona, Navarra, Spain; 3 Informatics Service, Clínica Universidad de Navarra, Pamplona, Navarra, Spain; 4 Center for Applied Medical Research, University of Navarra, Pamplona, Navarra, Spain; 5 CIBER-CV, Pamplona, Navarra, Spain

Venous thromboembolism (VTE) is a common complication in cancer patients, leading to significant morbidity, mortality, and resources consumption. Around 20% of VTE events are related with an underlying malignancy. 1 The incidence of cancer-associated thrombosis is increasing in recent years due to different reasons such as longer survival and improved sensitivity of imaging techniques. Hospitalization is a recognized additional risk factor. 2 Current evidence-based clinical practice guidelines (CPGs) uniformly recommend pharmacological prophylaxis with low-molecular-weight heparin (LMWH) in hospitalized cancer patients, unless contraindicated. 3 4 5 However, the quality of the evidence that supports LMWH prophylaxis in cancer inpatients is not strong as recommendations are based on the results of clinical trials involving medical inpatients with different conditions (not only cancer). 6 CPG statement applies to both, solid and hematologic malignancies, given that a similar VTE-associated risk has been reported. In fact, in the validated Khorana's risk assessment model, lymphoma is regarded as a high-risk tumor-site category. 7

In this article, we have evaluated the rate of thromboprophylaxis use and outcome in daily clinical practice in consecutive hospitalized cancer patients, focusing in the differences between patients with solid or hematologic cancer.

In this prospective cohort study, consecutive adult (≥ 18 years old) cancer patients, not receiving anticoagulant therapy, admitted in the Department of Oncology and Hematology at the University Clinic of Navarra were recruited. Patients undergoing bone marrow transplantation were excluded. The study was approved by the institutional Ethics Committee (P88/2013) and all patients signed an informed consent. The risk of VTE of all inpatients was automatically calculated by using an application of our electronic clinical history software following the PRETEMED score. 8 9 10 In this point scale, major risk factors such as active cancer, previous VTE, acute myocardial infarction, ischemic stroke with limb paralysis, decompensated chronic obstructive pulmonary disease, and thrombophilia were assigned a score of 3; congestive heart failure, chronic renal insufficiency/nephrotic syndrome, severe acute infection, lower limb cast, and prolonged bed rest were assigned a score of 2; and pregnancy/postpartum period, recent prolonged flight, lower limb paresis, estrogen therapy, thalidomide/lenalidomide administration, use of central vein catheter, obesity, age greater than 60 years, and smoking were assigned a score of 1. High risk of VTE was defined as a cumulative score of at least 4 points. According to our institutional thromboprophylaxis protocol, medical inpatients with high risk of VTE should receive 3,500 IU of bemiparin daily, unless contraindicated. The primary outcome of the study was the rate of thromboprophylaxis use. Secondary endpoints were the incidence of objectively confirmed VTE (events diagnosed in the first 24 hours after admission were excluded), major bleeding (according to the definition of the International Society on Thrombosis and Haemostasis), 11 and mortality during follow-up. Follow-up period starts from admission to 30 days after discharge. Categorical variables were expressed as frequencies and percentages, and quantitative variables as either mean (±standard deviation) or median depending on distribution. The chi square/Fisher's exact tests were used to compare proportions. Type I error was established in 0.05. Statistical analyses were performed using IBM SPSS Statistics (version 22.0, Chicago, Illinois, United States) software.

From April 2014 to February 2017, a total of 1,072 consecutive adult cancer patients were included, 217 (20.2%) of them with a hematologic malignancy. The main characteristics of recruited patients, according to tumor type, are shown in Table 1 . There were no differences between groups regarding age, gender, length of stay, or active treatment. Of note, 15.2% of patients with hematologic cancer presented thrombocytopenia less than 50 × 10 ^9^ /L, compared with 3.5% of those with solid neoplasm. According to the PRETEMED score, 93% of patients were considered as high risk. The rate of LMWH thromboprophylaxis during admission was 43.3 and 73.8% in patients with hematologic and solid cancer, respectively (risk difference: 0.30; 95% confidence interval [95% CI]: 0.23–0.38; *p* < 0.001). The proportion of patients with low risk of VTE receiving LMWH was similar inside each subgroup: 5 of 14 (35.7%) patients with hematologic malignancy and 48 of 69 (69.5%) patients with solid tumors. Exclusion of low-risk patients did not vary the overall results. A survey, limited to high-risk patients, revealed that in 40% of hematologic patients not receiving LMWH prophylaxis during admission, the reason to withhold it was the responsible physician's opinion that the patient's risk was not high. This number was markedly lower, 10%, for inpatients with solid malignancies.

**Table 1:** Clinical characteristics of recruited patients

	Total	Solid	Hematologic	*p*
*N*	1,072	855 (79.8%)	217 (20.2%)	
Age (mean ± SD)	62.1 ± 13.3	61.7 ± 13.3	63.4 ± 13.4	ns
Sex (male/female)	626/446	493/362	133/84	ns
Length of stay (days) (median, range)	5 (1–140)	5 (1–97)	5 (1–140)	ns
Cancer site ( *n* , %)				–
Colorectal	177 (16.5%)	177 (20.7%)		
Lung	161 (15.0%)	161 (18.8%)		
Gastrointestinal	96 (9.0%)	96 (11.2%)		
Gynecologic	80 (7.5%)	80 (9.4%)		
Pancreas	77 (7.2%)	77 (9.0%)		
Renal/Urinary	44 (4.1%)	44 (5.1%)		
Breast	42 (3.9%)	42 (4.9%)		
Prostate	37 (3.5%)	37 (4.3%)		
Other solid	141 (13.2%)	141 (16.5%)		
Lymphoma	136 (12.7%)		136 (62.7%)	
Myeloma	56 (5.2%)		56 (25.8%)	
Leukemia	21 (2.0)		21 (9.7%)	
Other hematologic diseases	4 (0.4%)		4 (1.8%)	
Metastatic disease ( *n* , %)	–	578 (67.6%)		–
Chemotherapy ( *n* , %)	880 (82.1%)	694 (81.1%)	186 (85.7%)	ns
Platelets <50 × 10 ^9^ /L ( *n* , %)	63 (5.9%)	30 (3.5%)	33 (15.2%)	<0.001
PRETEMED score (median, range)	5 (3–13)	5 (3–13)	5 (3–13)	ns
PRETEMED ≥ 4 points ( *n* , %)	989 (92.3%)	786 (91.9%)	203 (93.5%)	ns

Abbreviations: ns, nonsignificant; SD, standard deviation.

**Fig. 1:**
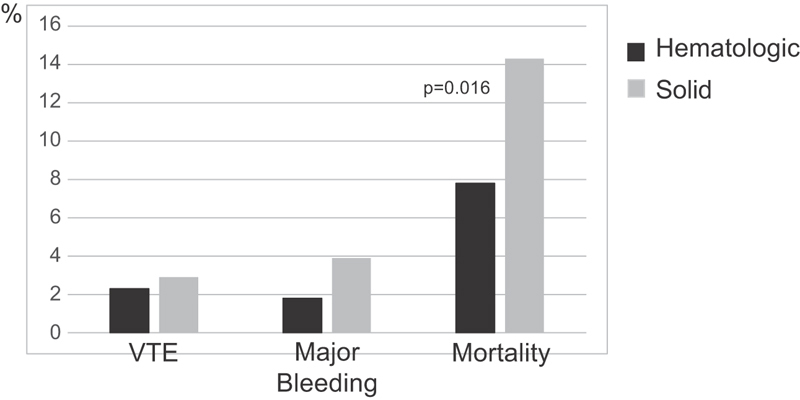
VTE events, major bleeding, and mortality during follow-up (hospitalization and 30 days after discharge). VTE, venous thromboembolism.

A total of 30 VTE events were observed during follow-up, 5 (2.3%) in hematologic patients and 25 (2.9%) in patients with solid cancer (relative risk [RR]: 0.79, 95% CI: 0.30–2.03; *p* = 0.79; Fig. 1 ). All the events developed in patients with high-risk score. Interestingly, in hematologic patients, four of the five episodes (80%) were catheter-related deep vein thrombosis (CR-DVT), and three of the five happened in patients not receiving prophylaxis with LMWH, one of them in a patient with a platelet count less than 50 × 10 ^9^ /L. In patients with solid cancer, 6 of 25 events (24%) were CR-DVT and 22 of 25 (88%) occurred despite appropriate thromboprophylaxis during admission. The rates of major bleeding during hospitalization and 30 days after discharge were low in both groups (1.8 vs. 3.9%; RR: 0.48, 95% CI: 0.17–1.33; *p* = 0.21). Gastrointestinal hemorrhage was the most frequent bleeding site in both groups of patients. Thromboprophylaxis was not associated with an increased incidence of major bleeding. Mortality during follow-up was 7.8 and 14.3%, respectively (RR: 0.55, 95% CI: 0.34–0.89; *p* = 0.016). Cancer progression was the leading cause of death, while bleeding and VTE were responsible for two and one deaths, respectively.

With the limitations of a single-center investigation, our study confirms a lower use of pharmacological thromboprophylaxis in hospitalized patients with hematologic malignancies compared with subjects with solid cancer. 12 However, only in a small subset of patients, there was a clear contraindication such as severe thrombocytopenia or active/recent bleeding. It appears that clinicians do perceive these patients as a lower-risk population, probably because many patients maintained some degree of mobility. 13 Nevertheless, in our study a higher number of hematologic inpatients showed very high scores in the PRETEMED scale. Unfortunately, we lack data about the use of extended prophylaxis after discharge, which could be necessary in very high risk patients but not routinely prescribed in most cases. 14 Although the incidence of VTE during or shortly after hospitalization is similar in both groups, some particularities exist. The absolute number of VTE events is low, but the higher proportion of CR-DVT in hematologic patients may be relevant because the effectiveness of LMWH for the prevention of that specific thrombosis site is controversial. 15 Currently, routine thromboprophylaxis for the prevention of CR-DVT in cancer patients is not recommended. 16 In addition, this different outcome in terms of thrombus location may imply that cancer site must be taken into account when designing future trials on VTE prevention in cancer patients. 17 Moreover, in our series, a trend toward a lower bleeding risk, not related with the use of thromboprophylaxis, and a significant lower short-term mortality was observed in hematologic patients. Specific studies in this population are warranted.
